# Machine Learning for Industry 4.0: A Systematic Review Using Deep Learning-Based Topic Modelling

**DOI:** 10.3390/s22228641

**Published:** 2022-11-09

**Authors:** Daniele Mazzei, Reshawn Ramjattan

**Affiliations:** Department of Computer Science, University of Pisa, Largo B. Pontecorvo 3, 56127 Pisa, Italy

**Keywords:** machine learning, industry 4.0, topic modelling, deep learning, systematic review

## Abstract

Machine learning (ML) has a well-established reputation for successfully enabling automation through its scalable predictive power. Industry 4.0 encapsulates a new stage of industrial processes and value chains driven by smart connection and automation. Large-scale problems within these industrial settings are a prime example of an environment that can benefit from ML. However, a clear view of how ML currently intersects with industry 4.0 is difficult to grasp without reading an infeasible number of papers. This systematic review strives to provide such a view by gathering a collection of 45,783 relevant papers from Scopus and Web of Science and analysing it with BERTopic. We analyse the key topics to understand what industry applications receive the most attention and which ML methods are used the most. Moreover, we manually reviewed 17 white papers of consulting firms to compare the academic landscape to an industry perspective. We found that security and predictive maintenance were the most common topics, CNNs were the most used ML method and industry companies, at the moment, generally focus more on enabling successful adoption rather than building better ML models. The academic topics are meaningful and relevant but technology focused on making ML adoption easier deserves more attention.

## 1. Introduction

Industry 4.0, or the fourth industrial revolution, expresses the rapid changes to the industrial world due to the combined improvements of technologies that join the physical and digital worlds [1]. These technologies refer to the inter-connectivity of the internet of things (IoT), robotics and edge devices, as well as the smart automation brought by artificial intelligence [2,3,4]. Considering the large scale of industrial problems, the proven success in scalability and automation of Machine Learning’s (ML) predictive power holds a lot of potential to thrive here. Hence, in recent years, researchers and companies are exploring ML for industry 4.0 more and more, seeking these benefits [5]. Bertolini et al. put forward a review discussing the applications of Convolutional Neural Networks (CNNs) and Autoencoders to industry problems [6]. Similarly, Gupta and Farahat presented a tutorial at the 2020 ACM SIGKDD Conference on Knowledge Discovery and Data Mining, highlighting new methods for industrial AI such as deep Reinforcement Learning (RL) [7].

However, industrial applications of ML are a complicated space due to the number of different intersecting domains, and the spike in interest over recent years, while positive, has made it challenging to thoroughly follow the trends of published work. A clear view of the area at scale allows interested parties to see evidence of rapidly increasing interest and, more specifically, where the attention of the community lies. This information is critical because it allows one to infer key points such as what research direction can be of useful contribution or what solution directions might be practical and worth real use.

For analysing and extracting useful insights from large datasets of text, natural language processing (NLP) techniques have shown positive results [8]. Wang and Zhang reviewed different means of recognizing method entities in academic literature using NLP [9]. Firoozeh et al. also examine keyword extraction methods as a means of extracting knowledge from large text datasets [10]. Keyword extraction is a powerful means of understanding what an entire corpus is about. Topic modelling methods, on the other hand, can count and cluster important words in order to identify the major themes within the corpus [11]. An example of this is seen in the work by Jacobi et al. where they apply a topic modelling technique, Latent Dirichlet Allocation (LDA), to a news corpus [12]. This approach allows one to discover topics of semantic similarity with richer depth and less manual input than using keyword extraction or simple statistical counts on the corpus.

This paper aims to provide a clear view of how ML methods intersect with industry 4.0 problems by analysing academic publications using NLP techniques. Through topic modelling, we were able to extract the main subareas of research from a dataset of scientific publications relevant to ML in industrial settings. Further analysis also allowed us to compare the use of ML techniques within each identified topic. Through these extractions, we answered the following research questions:

1. What are the industry 4.0 problems where ML solutions see the most discussion?
2. Which ML methods are used the most in these areas?
3. How do the areas focused on in the academic literature compare to the areas of focus in the white papers of top industrial companies?

Instead of a traditional manual review of papers, the focus of this review is on the automatic extraction of insights in the field from a large unreadable corpus of papers. However, brief descriptions of a subset of the well-known ML methods and industry 4.0 problems are still important for a thorough introduction. Hence, the remainder of the introduction section will highlight these areas, but the systematic review is not limited to them.

### 1.1. Machine Learning Methods

#### 1.1.1. Learning Paradigms

Before presenting specific methods, we must first clarify the major categories of learning paradigms they fall into.

1. Supervised Learning. This refers to methods that are trained using labelled examples. They can be highly accurate and trustworthy if the inferences made in real use are similar enough to the examples used during training.
2. Unsupervised Learning. This refers to methods that are used on unlabelled data. They are relatively less accurate but can be effective depending on the scenario and problem.
3. Reinforcement Learning. This refers to methods that reward positive or correct behaviour and punishes incorrect behaviour. The difference is clarified here as it does not fall under the aforementioned learning paradigms but more on this type of learning is discussed in its relevant section below.

#### 1.1.2. Neural Networks

An artificial neural network (ANN) is generally comprised of an input layer, one or many hidden layers of neurons and an output layer. An artificial neuron consists of input, weights applied to each input, a bias that is applied to the sum of the weighted inputs, and an activation function that converts the result to the expected form of the output (for example, the sigmoid function for a classification value of 0 or 1) [13,14,15].

A neural network with a single hidden layer is typically called a perceptron network, while networks with many hidden layers are referred to as Deep Neural Networks or Deep Learning and are at the core of many modern and popular ML methods [16,17]. The various ML techniques we are about to discuss are deep neural networks that use different structures of layers as well as specific mechanics relevant to their types of data and problems.

#### 1.1.3. Convolutional Neural Networks

Convolutional Neural Networks (CNNs) are one of the most popular and successful deep learning architectures. Although based on Neural Networks, they are mainly used in the field of Computer Vision (CV), for image-based pattern recognition tasks such as image classification.

Aside from input and output, the CNN architecture is typically composed of these types of layers: convolutional layers, pooling layers and fully connected layers. A convolutional layer computes the scalar product between a small region of the input image or matrix and a set of learnable parameters known as a kernel or filter. These calculations are the bulk of the CNN’s computational cost. The rectified linear unit (ReLU) activation function is also applied to the output before the next layer. A pooling layer performs downsampling, by replacing some output with a statistic derived from close information. This reduces the amount of input for the next layer, therefore reducing computational load. A fully connected layer is where all neurons are connected as in a standard ANN. This followed by an activation function helps produce scores in the expected format of the output (i.e., a classification score). Despite being computationally expensive, CNNs have seen many successful applications in recent years [18,19,20].

#### 1.1.4. Recurrent Neural Networks

Recurrent Neural Networks (RNNs) perform particularly well on problems with sequential data such as text or speech or instrument readings over time. This is because, unlike other deep learning algorithms, they have an internal memory that is meant to remember important aspects of the input. A feedback loop instead of forward-only neurons is what enables this memory. The output of some neurons can affect the following input to those neurons [21,22].

However, because of the vanishing and exploding gradient problems caused by the way the neurons affect many others through memory in RNNs, their ability to learn effectively becomes limited. Hence, the Long Short-Term Memory (LSTM) and Gated Recurrent Unit (GRU) methods aimed to solve this issue and rose to popularity as well. They do so by using gates to determine what information to retain [23,24,25,26].

#### 1.1.5. Support Vector Machines

Support Vector Machines (SVMs) are linear models that can be used for both classification and regression problems. SVMs approximate the best lines or hyperplane for separating classes by maximising the margin between the line or hyperplane and the closest data points [27,28,29]. Although this best-fit separator can be used for regression, it is more commonly used for classification problems. It is considered to be a traditional ML method compared to its deep learning counterparts but can achieve good results with relatively lower compute and training data requirements.

#### 1.1.6. Decision Trees and Random Forests

Decision trees are graphs comprised of nodes that branch off based on thresholds. They can be constructed by recursively evaluating nodes or features to find the best predictive features [30,31]. By itself, it can be used to make predictions, but to increase the performance of the method and mitigate overfitting, an aggregated collection of decision trees called a random forest can be used. Random forests as an ensemble learning method can accomplish this by training some trees on subsets of the data or features and aggregating the results [32,33]. The technique of training trees on different samples or subsets of data is called bootstrap aggregating or “bagging” [34].

They generally outperform decision trees but, depending on the data and problem, may not achieve an accuracy as high as gradient-boosted trees. Boosting is a technique where the random forest is an ensemble of weak learners or shallow decision trees that perform slightly better than guessing [35]. The intuition here is that weak learners are too simple to overfit and therefore their aggregated model is less likely to overfit. Gradient boosting builds on top of this by introducing gradient descent to minimize the loss in training [36,37]. An example of a popular and practical library implementation of gradient boosting is XGBoost [38].

Much like the aforementioned SVMs, algorithms based on decision trees are considered to be more traditional than deep learning methods and work especially well in situations with low compute and limited training data.

#### 1.1.7. Autoencoders

Autoencoders are ANNs that follow the encoder–decoder architecture. They aim to learn efficient encodings of data in an unsupervised way. The encoder is responsible for learning how to produce these lower dimension representations from the input, while the decoder reconstructs the encodings to their original dimensions [39,40,41]. Autoencoders are commonly associated with dimensionality reduction, as a deep learning approach to the problem traditionally handled by methods such as Principal Component Analysis (PCA) [42]. Reconstruction by the decoder can be useful for evaluating the quality of encodings, generating new data or detecting anomalies if performance significantly differs from normal cases. So, generally, some common applications of autoencoders include anomaly detection, especially in cyber-security, facial recognition and image processing such as compression, denoising or feature detection [43,44,45].

#### 1.1.8. Reinforcement Learning

Unlike the previously described supervised and unsupervised learning methods, Reinforcement Learning (RL) trains models by rewarding and punishing behaviour [46,47]. The intuition behind this is to let models explore and discover optimal behaviours instead of trying explicitly to train that behaviour with many samples. In RL, the model is defined as an agent that can choose actions from a predefined set of possible choices. The agent receives a sequence of observations from its environment as the basis or input for deciding on actions. Depending on the action chosen the agent is rewarded or punished for it to learn the desired behaviour.

This training is accomplished through defining concepts such as a policy, reward function and value function. A policy is a function that defines the agent’s behaviour, it maps the current observable state to an action and can be either deterministic or stochastic. A Value function estimates the expected return or reward of a certain state given a policy function. This allows the agent to assess different policies in a particular situation. The reward function returns a score based on the agent’s action in the environment’s state (i.e., a state–action pair).

Deep RL is attained when deep neural networks are used to approximate any of the prior mentioned functions [48]. Proximal Policy Optimization (PPO), Advantage Actor-Critic (A2C) and Deep Q Networks (DQN) are some examples of popular Deep RL algorithms. RL also sees successful practical use areas such as games, robotic control, finance, recommender systems and load allocation in telecommunications or energy grids [49,50,51].

#### 1.1.9. Nearest Neighbour

The Nearest Neighbour (NN) method is a simple algorithm that finds a defined number of samples closest to the new input point [52,53,54]. It is often used as a method for classifying new points based on the closest stored points, where closeness as a metric of similarity can be defined but is usually standard euclidean distance. Computation of the nearest neighbours can be conducted by brute force, or by methods devised to address brute force’s shortcomings such as K-D tree or Ball Tree [55,56]. Despite being such a simple method, NN has shown to be effective even for complex problems.

#### 1.1.10. Generative Adversarial Networks

Generative Adversarial Networks (GANs) are unsupervised models concerned with recognizing patterns in input data to produce new output samples that would pass as believable members of the original set. The GAN architecture consists of a generator, a DL model for producing new samples, and a discriminator, a DL model for discerning fake samples from real ones. The discriminator receives feedback based on the known labels of which samples are real and the generator receives feedback based on how well the discriminator discerns its output. Thus, the networks are trained in tandem [57]. Despite being the most recent of the discussed methods (first described in 2014), its adoption in real cases is growing rapidly given the high potential usefulness of generating data points to support meaningful problems with limited data availability. Direct applications aside from training data synthesis also include, among others, image processing such as restoration or superresolution, image-to-image translation, generating music and drug discovery [58,59].

### 1.2. Industry 4.0 Problems

#### 1.2.1. Fault Detection and Diagnosis in Maintenance

Considering the strong implications for safety, efficiency and cost, monitoring for machine malfunctions in an effective manner is a task that is both common and of high importance in the industrial world. Therefore, the scalable automation of these Fault Detection and Diagnosis (FDD) systems through ML techniques is one of the most popular types of ML applications in the field [60,61,62].

Given the nature of many faults such as signs of deterioration or surface defects on manufactured products, visual inspection is a regular and meaningful aspect of FDD systems. Hence, CNNs are regularly utilized in systems that aspire to automate this [63,64,65,66,67,68].

However, with the unique variety of the machines, products and components FDD systems must deal with, procuring large image datasets for each of them to leverage CNNs is no easy task. Time-series sensors that record metrics such as pressure, vibration or temperature are far more common in industry settings. So models that attempt to automate FDD through those relevant data types are also seen. With the success and popularity of CNNs, some still try to apply them to this data by using visualizations of the time-series data [69]. However, models that specifically focus on these data types such as RNNs, although not as commonly seen, are developed and deserve as much attention [70,71,72].

#### 1.2.2. Predicting Remaining Useful Lifetime

In a similar vein to FDDs, the efficiency and planning of industry maintenance can be empowered by predicting how much useful time a machine or part has left. This can be applied to components that need regular replacement such as bearings or batteries [73,74], or in more challenging cases, can be sudden rapid failures in complex processes such as ion milling [75]. With the stronger temporal aspect to this problem, sequence models are more commonly seen [75,76,77] but creative approaches using other methods such as autoencoders [78], decision trees [79], RL [80] and GANs [81] have also been presented.

#### 1.2.3. Autonomous Operation

Automation of repetitive operations is another impactful area for leveraging ML in industry 4.0. Robotic operation is one of the most direct approaches to this and most commonly makes use of CNNs or RL or both [82,83,84]. RL is an effective method for developing agents or models that are dynamic and adaptable to the same tasks in different environments, while CNNs are useful here for recognizing visual features in tasks such as object detection or aiding RL agents in observing the environment space. These applications can be as generic as automated pick and place [85] or as use-case specific as, for example, automating coastal cranes for shipping containers [86]. UAV navigation for smart agriculture is also another strong growing application worth mentioning [87,88].

However, autonomous operation also reaches other aspects of industrial businesses such as customer service or marketing operations [89,90]. As these domains are inherently more digital, they are a source of simpler and easier but still effective forms of automation.

#### 1.2.4. Forecasting Load

Load forecasting refers to predicting changes in demand over time, typically regarding energy or electrical grids. Load forecasting is critical for adequate preparation to cater for increased demands. For example, consider a power company that may need to acquire additional materials such as natural gas to meet the expected demands. Accurate forecasting will not only result in better services but can significantly improve economic and environmental efficiency in their energy usage [91,92].

As a temporal regression problem, this industry 4.0 use case is one of the few where deep sequence models such as RNNs or LSTMs are used much more than alternative models [93,94,95]. Furthermore, while there is evidence that shows they do perform well, they are not exempt from common industrial challenges in adoption and practical use such as data availability or complex system integration.

#### 1.2.5. Optimizing Energy Consumption

The case of optimizing energy consumption in industrial settings, in some ways, can be viewed as an extension of the previously described load forecasting problem. There is also some similar usage of ML, in that models are often designed for forecasting energy consumption [96,97].

These forecasts can be useful in supporting decisions to optimize the response to that demand. An example of this is seen in work conducted by [98], where they optimize demand responses through a rule and ML-based model for controlling and regulating a heat pump and thermal storage. Similarly, in [99], IoT-based data collection was used to optimize energy consumption for a food manufacturer by providing them with analysis results to support decisions. Other examples of specific approaches to optimizing energy consumption include offloading ML compute to the edge or fog [100], using RL for optimizing the trajectory of UAVs in intelligent transportation systems and smart agriculture [101,102] and using the ant colony optimization algorithm to improve routing performance and therefore energy consumption in wireless sensor networks [103].

#### 1.2.6. Cyber-Security

One of the most general and common use cases faced in industry 4.0 regardless of the specific field is Cyber-Security. As digitization increases more and more so does the need to sufficiently protect those digital assets and processes. The importance and priority of security are also notably higher for supervisory control and data acquisition (SCADA) systems [104]. This is because SCADA is a category of applications for controlling industrial processes and therefore a digital interface to large-scale physical components. Furthermore, the historic ramifications of famous attacks such as Stuxnet act as evidence of the threat and dangers posed by poor security practices [105].

Malicious attacks can be viewed as very unusual behaviour the system does not expect in regular use, and because of this, from an ML standpoint it is often formulated as an anomaly detection problem. Traditional methods such as k-Nearest Neighbours-based clustering algorithms or decision trees can be used to approach this problem [106,107,108], but in recent years deep autoencoders have seen a lot of successful use [109,110,111,112]. This is performed by training on data, such as activity logs or network requests, that is almost all normal and non-malicious. If the malicious activity goes through the autoencoder then, because it is anomalous and unlike previous data, the decoder would reconstruct it more poorly than usual.

It must be noted however that although the formulation of anomaly detection is effective and popular, it is not perfect in the case of complex attack sequences trying to mimic normal behaviour. To that end, other methods for security and intrusion detection are still just as important. For example, RL has also seen use in vulnerability analysis by seeking to train agents to be both attackers and defenders for the system being evaluated and learn complex attack behaviours [113,114,115].

#### 1.2.7. Localizing Assets

While the Global Positioning System (GPS) is often sufficient for determining location in outdoor environments, the indoor localization of assets is a more challenging problem due to the lack of line of sight to satellites. This is useful for several applications including security by tracking entry to unauthorized areas, factory safety by ensuring the right need number of people is maintained and data analytics by providing an additional means of monitoring processes and key performance indicators (KPIs) such as idle time or loading time [116].

For indoor localization Wi-Fi fingerprinting has become one of the most common technology choices as it does not require a line of sight and can work with any Wi-Fi-capable device without any additional hardware [117]. Deep learning has successfully supported this area by enabling cases such as self-calibrating fingerprint databases for localization with autoencoders [118] or recognizing received signal strength (RSS) patterns in device-free localization with autoencoders and CNNs [119,120].

#### 1.2.8. Soft Sensors

The complexity of industrial processes, especially in cases such as the chemical, bioprocess or steel industries, is often reflected in a large number of sensor metrics and variables for monitoring, controlling and optimizing these processes. Soft Sensors are software-based data-driven ways to estimate, simplify and model these large numbers of physical sensors with varying constraints [121,122]. By having this representation of the process’s state, it becomes easier to detect faults, recognize changes in performance through tracking and optimize decisions in scheduling or supply chain management. Traditional statistical methods such as PCA or support vector machines (SVM) have often been applied to soft sensing [123], but modern methods such as autoencoders that can produce latent representations of data well have also seen use [111,124].

#### 1.2.9. Logistics and Resource Allocation

The efficiency of logistical issues such as delivery schedules, manufacturing pipelines, raw material management and the allocation of resources throughout all processes are incredibly important to lowering costs and maximizing productivity in industrial companies [125,126], while these issues are still handled quite well by optimization algorithms such as particle swarm or ant colony optimization [127,128,129,130,131]. There has been increasing interest and usage of RL for these problems [132,133]. RL and its exploratory behaviour-driven means of solving problems can allow for greater flexibility in adapting to unforeseen circumstances, especially in the case of the ever-changing and unique needs some companies may have. That being said, RL solutions are complex to develop and implement as is, this becomes even more challenging when companies must find a way to integrate them into their already complex processes so optimization algorithms still stand as the stronger simpler source of solutions.

## 2. Related Works

There have been several reviews and surveys in the space of ML for industry 4.0. Some focus on specific ML application areas such as predictive maintenance [60,134,135], soft sensing [136] and fault detection [137]. Some try to be more comprehensive, looking at ML applied to an entire industry or common pipelines, such as manufacturing [138,139,140,141,142], transportation [143,144] and energy systems [145,146].

While others, in a similar vein to this paper, aim to cover the entire area of ML for industry 4.0. For example, the tutorial by Gupta and Farahat exemplified impactful industrial applications based on categories of ML methods [7]. Similarly, work in [142] and [147] provide an overview of how ML methods can enhance solutions to industrial problems. However, although a review based on manually read papers can provide an in-depth analysis, they are limited to amounts that can be feasibly read and only observe a limited sample of the industry 4.0 literature. The aforementioned reviews are useful and impactful works, but cannot provide insight on some questions, such as what industrial business functions receive the most attention or which receive too little, without quantitative results.

Hence, systematic reviews of this nature have also been explored, for example by Bertolini et al. [6]. They first curated a dataset of papers by querying the Scopus, Web of Science and Google Scholar databases. They then performed a series of restrictions to refine the dataset down to 147 papers which they manually reviewed. A similar approach was taken by Liao et al. by manually vetting papers included in their analysis [148]. Such an approach can extract highly relevant and representative papers for detailed insights, such as key applications and techniques, through manual review.

Even so, larger-scale insights can be attained by working with the bigger datasets that are possible given the massive trustworthy databases available. Lee and Lim explore an industry 4.0 review based on text-mining and provided insightful clarity on the state of the field [149]. Nonetheless, their method was only semi-automated and included a limited dataset of 660 articles up to 2018. Advanced NLP methods, specifically Topic Modelling, enable the automated analysis of large-scale document sets that are infeasible for manual reading. The effectiveness of Topic Modelling for analysing research fields was exemplified by the work of Mazzei et al. surveying Social Robotics [150] and Atzeni et al. observing ML and Wi-Fi [151]. This approach can be useful for understanding the space at large by allowing the insights to be truly data-centric rather than heavily influenced by the sampling method. That is, its benefit over manual reviews is that it can cover a vast number of publications, infeasible for manual reading, and discover its topics. To the best of our knowledge, at the time of writing, there are no systematic reviews such as this for ML in industry 4.0.

## 3. Methodology

This section will detail the steps behind obtaining, preparing and analysing our data with respect to our goals and previously discussed approach. We break down the methodology into the steps of paper gathering, preprocessing, meta-analysis, topic modelling and topic analysis.

### 3.1. Paper Gathering

**Figure 1:**
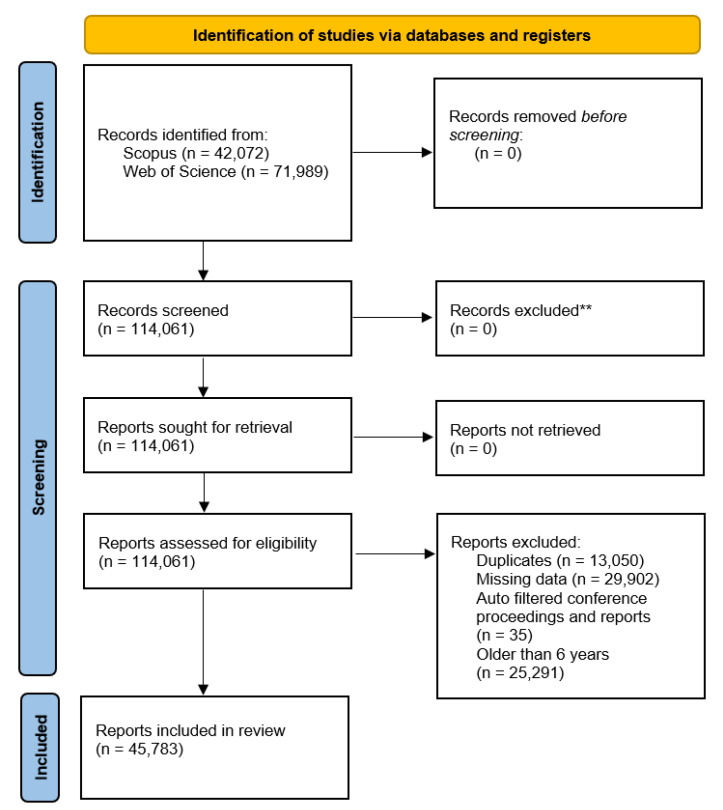
PRISMA flow diagram of dataset curation. ** Count includes exclusions by both humans and automated tools.

Our dataset curation follows the structure provided by the PRISMA statement for reporting on systematic reviews. Figure 1 illustrates the structure followed. Note that all of the report screening was completed after retrieving the data due to the limitations of the database APIs used.

The papers retrieved for this study were sourced from Scopus and Web of Science via their respective APIs. These databases are commonly used for systematic reviews and their comprehensiveness has been studied [152,153]. The query presented in Listing 1 was used for both sources. The query constrains results to mention both a term referring to industrial settings as well as a term strongly relevant to machine learning or some of its most popular methods. Due to the limitations of the database providers, only paper titles, abstracts and metadata were collected.

**Listing 1.** Paper Retrieval Query.
(‘industrial’ OR ‘industry 4.0’)
AND
(‘deep learning’ OR ‘machine learning’
      OR ‘artificial intelligence’ OR ‘convolutional neural’
      OR ‘recurrent neural net’ OR ‘long short-term memory’
      OR ‘deep autoencoder’ OR ‘reinforcement learn’
      OR ‘generative adversarial network’ OR ‘deep neural’)


Scopus returned 42,072 papers and Web of Science returned 71,989. After removing duplicates, the dataset had 71,074 papers with 21,283 and 49,825 coming from Scopus and Web of Science, respectively. We then restricted the dataset to papers from the most recent 6 years because changes in the trends of ML and data analytics are rapid, and we are more interested in the currently prevailing topics than those of a decade ago.

**Figure 2:**
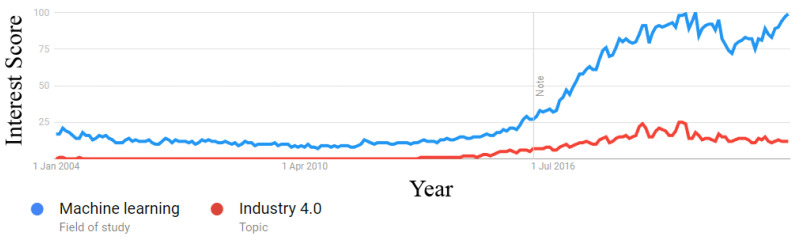
Google Trends’ interest over time for Machine Learning and industry 4.0.

**Figure 3:**
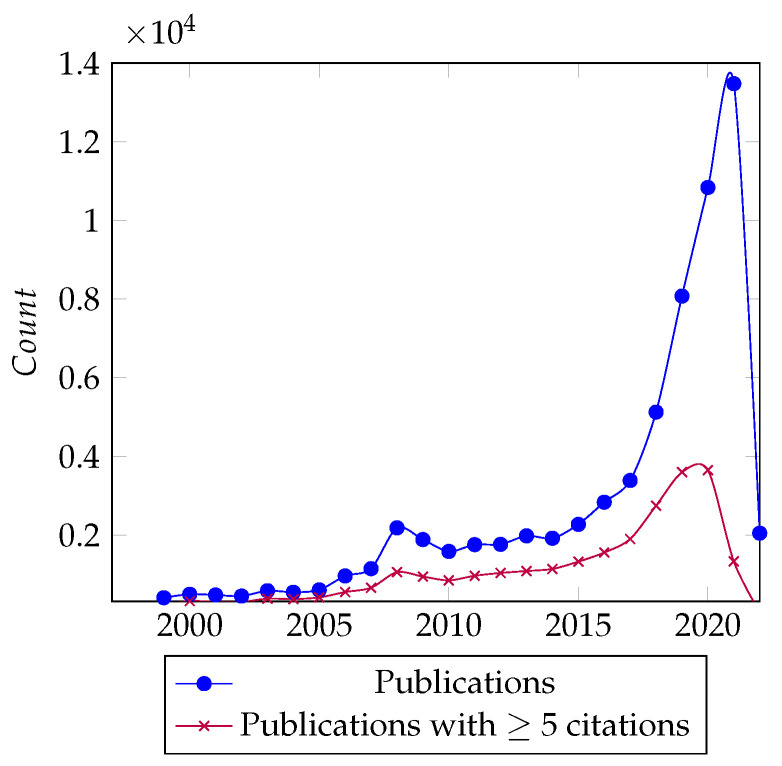
ML in industry 4.0 publications and citations since 1999.

Figure 2 shows Google Trends’ interests over time for Machine Learning and industry 4.0. There was a substantial increase in interest for both topics beginning in January 2016. Furthermore, Figure 3 shows the publications over time from the initial papers retrieved, and there is a clear spike in the number of papers from 2016 onward. These observations further support our decision to restrict our analysis to the last 6 years. Hence, the final corpus consisted of 45,783 papers from January 2016 to February 2022.

### 3.2. Preprocessing

Data cleaning tasks firstly consisted of catering for the differences in the fields returned by the two databases in order the merge paper sets for analysis. Secondly, the main text corpus to be analyzed was prepared. This included the following: combining the title, keywords and abstract fields, converting all characters to lowercase, lemmatization and lastly, removing punctuation, digits and stopwords.

### 3.3. Meta-Analysis

The preliminary analysis was aimed at supporting our attempt to answer the research questions targeted in Section 1. This meta-analysis included: a plot of papers over time, a count and comparison of paper source types, and counts of papers that directly reference key popular ML methods.

### 3.4. Topic Modelling

The topic modelling performed on the corpus utilized the BERTopic algorithm [154]. This technique was chosen over others such as LDA [155] or NMF [156] because BERTopic requires less effort in hyperparameter tuning and it trampolines off of the successful transformer-based model, BERT [157]. It also empirically showed better results in topic coherence and topic diversity on benchmark datasets [154].

As described in its original article, the BERTopic algorithm comprises the following major steps:

1. **Paper Embeddings with BERT**. Converting the text of the input papers to a numerical representation is the first step. BERT is used for this because it extracts embeddings according to the context of the words and the number of available pre-trained models makes it easier to obtain more accurate extractions. The Sentence-BERT implementation and pre-trained models are commonly used and were used in this case as well [158].
2. **Embedding Dimensionality Reduction with UMAP**. Before clustering the embeddings to discover topics, dimensionality reduction is performed using UMAP because many clustering algorithms perform poorly on high-dimension data. UMAP was chosen because of its good performance in retaining information [159].
3. **Paper Clustering with HDBSCAN**. With the dimensionality reduced to a reasonable amount, the embeddings are then clustered. HDBSCAN is chosen by the author because it does not force data points into clusters. It instead considers them outliers and it works well with UMAP since UMAP maintains structure well even in a low dimensional space [160].
4. **Topic Representation with c-TF-IDF**. For deriving important representative words for the clusters of documents, a class-based variant of TF-IDF [161] that generalizes the method to a group of documents is used. Thus, resulting in a list of words representing a topic for each cluster. This representation is also used to give greater control over the number of clusters by merging similar and uncommon topics.

### 3.5. Topic Analysis

Upon the generation of the topic words by the prior step, we assessed the quality of the result by observing approximate visualizations of the clusters and manually vetting small random samples of each topic. If the quality of the result was deemed subpar, we adjusted the HDBSCAN hyperparameters, such as min_cluster_size (the minimum size of clusters) and min_samples (how conservative the clusters should be when determining outliers), and repeated the process. The topic labels were manually determined based on the produced topic words and the sample vetting. We then analyzed each topic to observe: the percentage present in the corpus for each topic, the counts of papers referencing important ML methods within each topic and the keywords by count for each topic.

### 3.6. Garnering an Industry Perspective

Our third research question sought to examine a comparison between the areas focused on in the academic literature versus those in the white papers of top industrial companies. To that end, we manually gathered a small sample of such white papers and extracted the key ML for industry 4.0 topics. The query, “[company] white papers industry 4.0 machine learning ai”, was used on a popular search engine, Google, for a brief list of top professional consulting companies. The blogs and websites of these companies were also directly checked. The companies and their works included McKinsey & Company [162,163,164,165,166], Accenture [167,168,169], Microsoft [170,171], Bain & Company [172], Deloitte [173], PriceWaterhouseCoopers [174,175] and Boston Consulting Group [176,177,178]. The papers were manually vetted and selected for a similar degree of relevance and recency to our academic corpus, resulting in a set of 17 white papers. These papers were not included in the academic paper corpus or analyzed using the topic modelling procedure, they were manually reviewed and their main ML for industry 4.0 topics were extracted. The topics were extracted if the authors considered them to be of significant potential value to industrial companies.

### 3.7. Meta-Analysis Results

**Figure 4:**
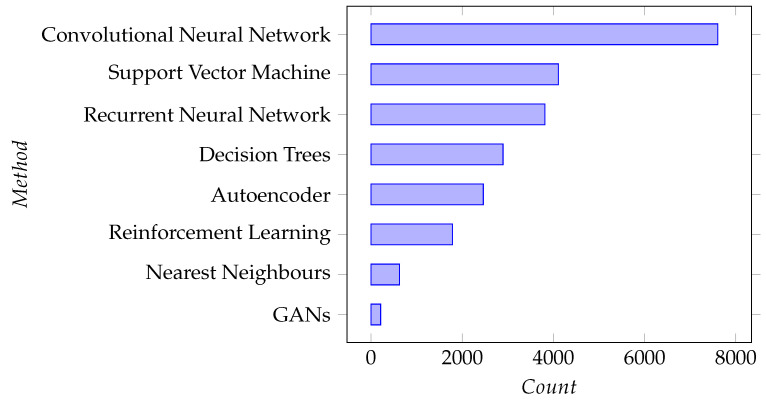
ML Methods by paper count for corpus.

A comparison of paper source types is presented in Table 1. Additionally, to gauge the presence of common ML techniques, we counted the papers that mention key popular ML methods in their title, abstract or keywords. This was conducted by writing lists of identifying terms for each ML method considered. The counts of papers referencing these ML methods are shown in Figure 4; however, “Neural Networks”, with a count of 36,229 papers, were excluded from that chart as it would overshadow the other results and affect readability.

## 4. Results

### 4.1. Topic Modelling Results

**Figure 5:**
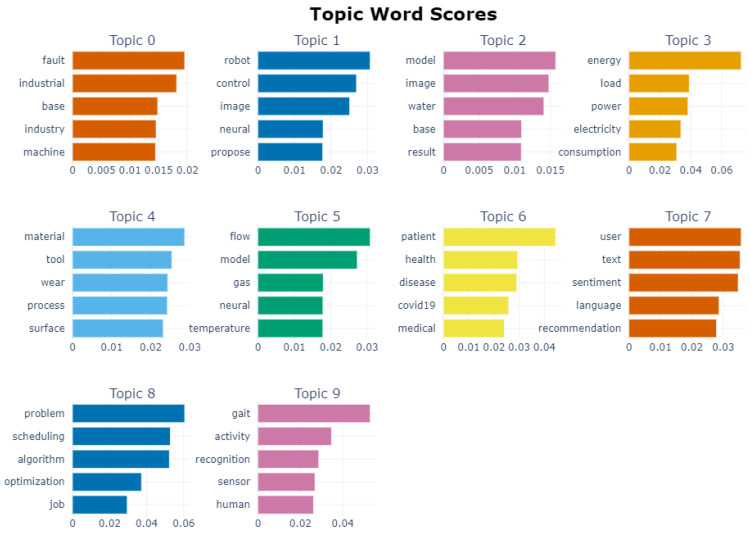
Plots of topic words by c-TF-IDF scores from topic modelling entire corpus.

**Figure 6:**
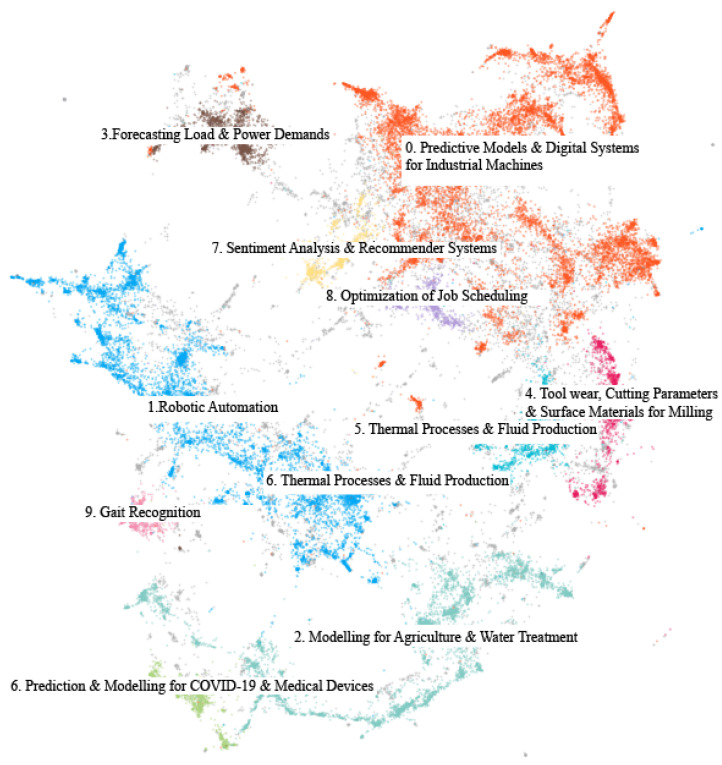
Visualization of topic clusters by further reducing embedding dimensions.

Figure 5 shows plots of the topic words and c-TF-IDF scores produced by the topic modelling model after tuning the clustering hyperparameters. By further reducing the dimensions of the embeddings to two during the UMAP step, we produced a visualization of the topic clusters as shown in Figure 6. This visualization as well as the sample vetting described in Section 3.4 allowed us to confirm that the topics cover the majority of the papers aside from outliers and capture their main themes. Based on reviewing the full lists of topic words and samples for each topic, the labels presented in Table 2 depict the primary topics found and their percentage of presence in the dataset.

However, with the size of our corpus, the top 3 topics are wide-spanning branches of the ML for industry 4.0 subfields. They also encompass several thousands of papers, which helps emphasize the significance of those areas but impede our ability to examine specific research directions. Hence we further analyzed the top three results by repeating the topic modelling, tuning and labelling processes on them to produce their sub-topics, while considering sub-topics, the top 20 topics are put forward in Table 3.

**Figure 7:**
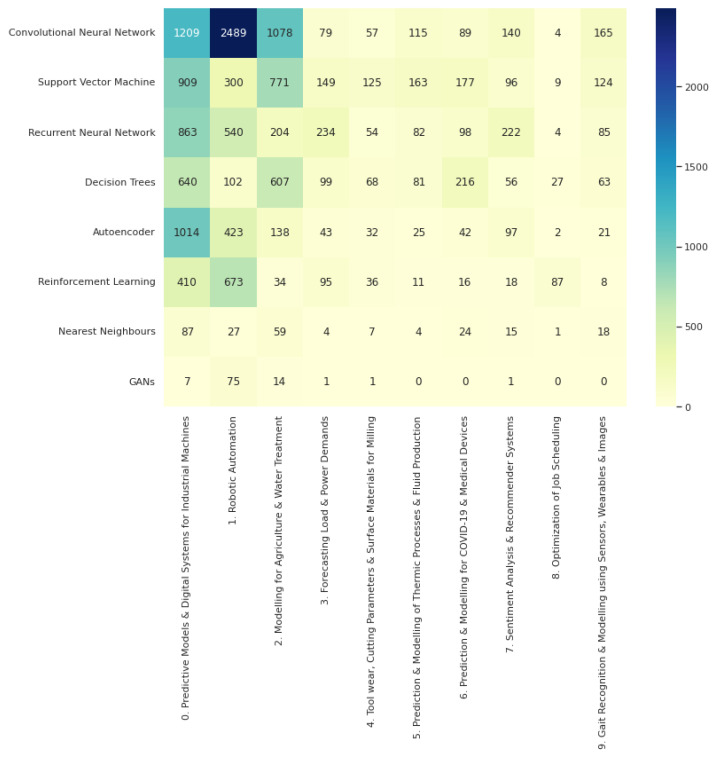
Count of papers by ML term mentions for each topic.

Furthermore, the counts by paper for ML methods were repeated for each topic. The resulting counts are reported in Figure 7.

### 4.2. Industry Perspective Results

**Figure 8:**
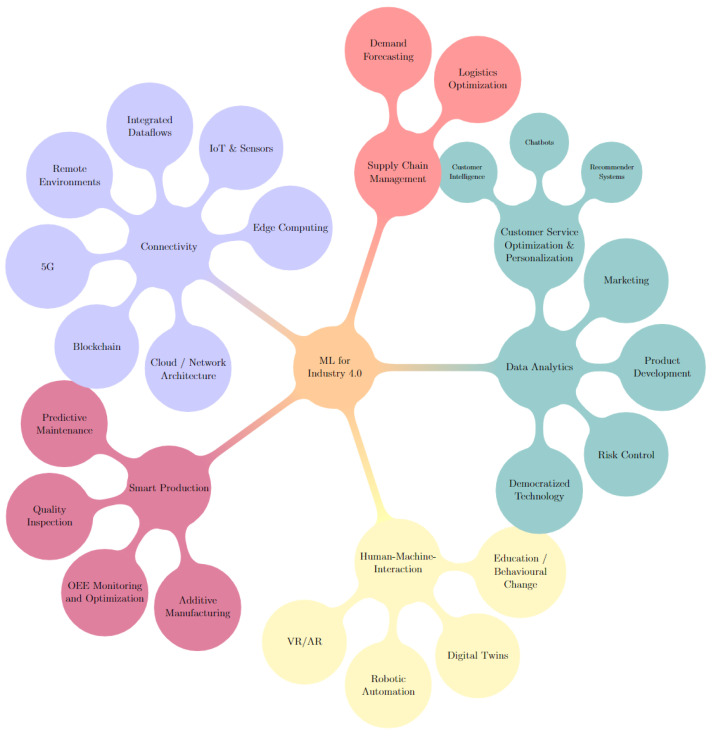
Mind map of high potential value areas for ML in industry 4.0, by mentions in top consulting companies’ white papers.

By manually reviewing the previously mentioned collection of white papers from consulting companies, we curated lists of high potential value areas in ML for industry 4.0 for each paper. These areas or topics were categorized, grouped and visualized in the mind map illustrated in Figure 8.

## 5. Discussion

### 5.1. Meta-Analysis

The meta-analysis results do not all directly contribute to answering our research questions, but they provide useful context on the state of the area and our data. The publications over time show the increasing interest in the area of ML for industry 4.0, with a strong spike in the last 6 years. By looking at the trend of publications with five or more citations, we see that the spike’s significance is less than 30% of its counterpart. This can be attributed to recency and the suddenness of the spike leading to many similar works competing. However, the trend of the spike in interest is maintained and allows us to estimate if the trend holds for impactful or popular papers.

The paper sources of our corpus are dominated by articles and conference papers, an expected result. Unexpectedly, the percentage of articles outshines the conference papers, a counter-intuitive result since Computer Science research findings tend to be published in conference papers [179]. Examining the results further we saw that for Scopus, the percentage of conference papers and articles were 67.9% and 25.5% respectively, while for Web of Science it was 20.8% and 73.3%. A likely contributor to this is that Scopus has better coverage of conference material, as previous work by Pranckutė has shown [180]. Hence, considering the Web of Science results outnumbered Scopus 33,632 to 12,151, it skews the final counts. Additionally, ML for industry 4.0 is much more interdisciplinary than the typical Computer Science sub-field so the tendency may not hold as well as usual.

### 5.2. RQ 1: What Are the Industry 4.0 Problems Where ML Solutions See the Most Discussion?

Towards answering this question we used topic modelling to extract insights from a large corpus and rationalized the choice of a deep learning-based approach in BERTopic. The modelling produced the top topic words for each cluster of papers, and we used the top 10 topic words in addition to manually vetted samples of clusters to assign final topic labels. The word scores in Figure 5 represent a quick look at what BERTopic produced. The topic words in that figure are the most common words in the topic defined by the algorithm. By themselves they clearly hint to what the topic is about, but to give accurate labels to each topic we also manually vetted random sample papers from each. The 2D visualization of the labelled corpus shown in Figure 6 makes it clear that the topics covered the corpus sufficiently with reasonable clusters. The clusters are cleanly separated and illustrate the differences in topic presence at a glance.

From Table 2 we can see that the top three topics are wide branches of the overall area. It is a useful observation to see their dominance at a general level but a closer inspection was deemed appropriate to meet the specificity of the remaining topics for a fairer comparison. The remaining 7 of that table were more specific cases of ML but the top 10 encapsulate the variety of the problems discussed in ML for industry 4.0.

Topics 0 and 1, “Predictive Models and Digital Systems for Industrial Machines” and “Robotic Automation”, are fairly general areas but show a significant focus on smart production applications. Topic 2, “Modelling for Agriculture and Water Treatment”, was a less expected observation. Smart agriculture is home to large branches of ML applications such as the classification of plants and crop health or using soil characteristics to inform decisions and actions. Hence it is understandable as a key topic. Water treatment on the other hand is a more specific class of industrial applications. The grouping of the two together is likely around the term “water” itself and can be construed as the model failing to distinguish the difference based on the implied context. This is a known limitation of BERTopic, where the topic representation stage is applied to bags-of-words and does not explicitly leverage the embedded representations in this step. This issue in addition to how wide of an area the top 3 topics cover, motivated further analysis on these three subsets by repeating the topic modelling procedure.

That process resulted in Table 3 where we see a similar degree of specificity across the topics. The general result of smart production being the most significant remained, but now we gain greater insight into the observation. Security and intrusion detection was the most prevalent area. taking into account the high potential costs and damage of cyber-attacks, the risks taken on by increasingly digitized systems and the regulatory compliances companies must meet, it is a logical finding that security is the most studied topic in the area. Similarly, another of the top 20 is gait recognition, a biometric authenticator often used as an added physical security measure [181]. Forecasting load and power demands, as well as optimization of job scheduling, are ultimately concerned with the goal of dynamically improving logistic processes in the supply chain. Sentiment analysis and recommender systems are a part of optimizing and personalizing customer service and are the only topic in the table concerned with this business function. The general theme of the remaining top 20 topics is of automating smart production tasks. Noteworthy inclusions among them, are “Fault diagnosis and detection” and “Predictive maintenance and RUL forecasting”. These are both focused on automating tasks that reduce the downtime of machines and are frequently a dominant topic in manual reviews.

### 5.3. RQ 2: Which ML Methods Are Used the Most in These Areas?

In a step toward answering the question “Which ML methods are the most common in the area?”, we counted the papers mentioning certain popular methods in their title, abstract and keywords. Across the entire corpus, Convolutional Neural Networks (CNNs) were the most common, with almost double the count of the second most common method. CNNs stand among the most popular deep learning methods given the recent string of successes in the computer vision domain. If we are to discuss how appropriate the choice of this model type is we must also consider the type of data most common in industrial settings for the problems many seek to solve. We cannot deduce this across the entire corpus easily, so we also look at the same count applied to each topic cluster of papers.

From Figure 7 we can see the method counts per topic. Convolutional neural networks (CNNs) are dominant in the top three topics but not by the magnitude the previous count figure alluded to. The clearest gap in usage is for the robotic automation topic. This is likely due to a combination of how much image data is present in that space and the popularity of computer vision applications in general.

Reinforcement learning (RL) is the second most popular for topic 1, “Robotic Automation”, which is interesting because the practical applications of this area are not as solidified as some of those in supervised learning. That result adds to the argument that robotic automation in industry 4.0 is a prime area for impactful real-world use of RL. This topic also has a higher than usual count for generative adversarial networks (GANs), which suggests that the space is often looked upon for the newer but exciting machine learning techniques. RL also has the highest count for the “optimization of job scheduling” topic but more traditional optimization techniques not covered in the scope of this review are more likely to be the standard solutions to this problem.

Recurrent neural networks (RNNs) see higher counts in topics where sequential or time-series data are more prominent, such as topic 3, “Forecasting load and power demands”, and topic 7, “Sentiment analysis and recommender systems”. However, it can be argued that for topic 0, “Predictive Models and Digital Systems for Industrial Machines”, one might expect to see RNNs over CNNs due to the heavy presence of multivariate time-series data in industrial machine sensors. The fact that autoencoders see a much higher count there than anywhere else attests to this. Thus, CNNs may be seeing more common use due to their popularity.

Meanwhile, the more traditional methods, support vector machines (SVMs) and decision trees see consistent mentions across all topics, likely due to their simplicity, lower computational demands and well-established reputations in the space of machine learning.

### 5.4. RQ 3: How Do the Areas Focused on in the Academic Literature Compare to the Areas of Focus in the White Papers of Top Industrial Companies?

Answering this question required a look at ML for industry 4.0 from the high-level perspective of top companies. To that end, we reviewed the recent and relevant white papers of top consulting companies to provide a foundation for comparing the academic literature’s focuses. We chose top consulting companies as they are often the ones providing guidance and insight to industry actors directly. These consulting companies can be considered leading experts in their practical domains, they also have an incentive to share their insights and trends publicly. The mind map shown in Figure 8 was the result.

If we categorize the topic modelling results presented similarly, each of the major categories in the mind map, such as smart production or connectivity, would be represented, while not every minor category, such as marketing or Virtual and Augmented Reality (VR/AR), is present in the topics extracted, this is understandable considering we look only at the top 20 specific topics. Moreover, some areas are inherently less “publishable” than others. For example, if a team were to discover a competitive edge in Product Development, publishing those findings could reduce that edge or eliminate it. Similarly, some areas provide more opportunities to publish by having a plethora of valuable use cases where ML models can be applied. Robotic automation and predictive maintenance are examples of such areas.

A limitation of the mind map is that it does not consider comparisons between the topics it covers when in reality not all of the high-potential areas shown are equal in impact and development complexity. So to gauge these aspects for our comparison, we also look at the specific results of the McKinsey & Company global survey on the state of AI in 2021 [166]. They surveyed 1843 participants, representing a full range of regions, industries, company sizes, functional specialities, and tenures, on their adoption and usage of AI. The survey shows that manufacturing use cases had the highest impact on decreasing costs. Hence, it makes sense that the academic literature would show a significant focus on smart production areas as well. Likewise, “Sentiment analysis and recommender systems” may seem like an inconsistent result among the other topics, but Customer Care and Personalization falls under Service Operations which, according to their survey, is the most commonly adopted AI use case category.

From this, we can posit that the academic literature generally aligns with the major focuses of industry experts. However, despite the aforementioned caveats, we believe that some areas still deserve more attention. Companies are focused not only on individual problems or use cases but also on the bigger picture of how they connect to the rest of their pipelines and how they integrate with existing systems. Therefore, we believe it would be worthwhile for future works to reflect this. Topics that lean towards this goal include democratized technology, Human–Machine-Interaction through digital twins and VR/AR, risk control concerning AI and ML in marketing.

## 6. Limitations and Promising Directions

One of the major limitations standing in the way of practical ML advancements that can help close the gaps between industry and academia is the availability of relevant data. Useful datasets certainly exist but given the variety of sources that occur from industry to industry, it is difficult to find truly comprehensive public datasets. The use of GANs to generate training samples for problems with limited data is therefore an interesting and useful direction of work, especially considering the large amounts of data DL methods require.

Another key issue is the complexity of deploying and integrating new ML systems. Large companies are likely to have capable teams and additional infrastructure in place to properly facilitate ML, but the vast majority of small to medium enterprises (SMEs) that do not have this would need to make an upfront investment for engineers or consultancy to explore the benefits of ML to their specific cases. Democratized technology and smart integration through IoT show great promise for simplifying the path to ML adoption. They enable general but useful ML solutions to be tried and built upon instead of upfront cost, thus smoothening the rate of investment required by SMEs.

Computer vision solutions have seen a lot of success and popularity in industry 4.0 and the attention it receives is well deserved. However, models that revolve around common existing sensors for machines (temperature, pressure, vibration etc.) and software (ERP, CRM, MES, etc.) would likely be cheaper in terms of computation and hardware. Therefore, time-series ML models relevant to these rich data sources are also a promising direction for advancing ML in industry 4.0.

## 7. Conclusions

Machine learning has a lot of potential value in industry 4.0 due to its scalable automation. This notion is supported by the spike in relevant publications over the last six years. With that much research activity, comprehensive reviews are needed to provide a foundation for guiding new studies and industry action plans, while there are several high-quality reviews in the field, not many attempt to review the area on a large scale and none utilize Topic Modelling to maximize the literature coverage. We aimed to do such a review by gathering papers from the Scopus and Web of Science databases, building a topic model using BERTopic, analysing the results and comparing it to a manually reviewed industry perspective.

We targeted our research towards three research questions, “What are the Industry 4.0 problems where ML solutions see the most discussion?”, “Which ML methods are used the most in these areas?” and “How do the areas focused on in the academic literature compare to the areas of focus in the white papers of top industrial companies?”. From reviewing the top 10 topics, we found that the most frequent problems fell under Security, Smart Production, IoT Connectivity, Service Optimization, Robotic Automation and Logistics Optimization. By counting the mentions of ML methods for each topic, we saw that CNNs were the most dominant despite the high presence of time-series data in industrial settings. We manually reviewed 17 company white papers to garner an industry perspective and compared them to our topics extracted from academic literature. In comparing the two, we observed that the coverage of areas generally aligned well, and the higher presence of smart production topics was justified given its real-world impact and the fact that some areas are more easily publishable than others.

However, we also recognized that companies are focused on higher-level goals rather than just individual ML use cases or improvements. Hence, we remarked that the topics supporting ML adoption and integration deserve attention and increased focus in future works. Examples of these areas include democratized technology, digital twins, human-AI-interaction and AI risk control.

## Figures and Tables

**Table 1:** Paper sources for corpus.

Source Type	Count	% of Data
Article	27,767	60.65
Conference Paper	15,664	34.21
Review	1496	3.27
Book/Chapter	504	1.1
Other	352	0.77

**Table 2:** Top 10 results of topic modelling.

Topic	% of Data
0. Predictive Models and Digital Systems for Industrial Machines	25.44
1. Robotic Automation	16.33
2. Modelling for Agriculture and Water Treatment	10.45
3. Forecasting Load and Power Demands	3.25
4. Tool wear, Cutting Parameters & Surface Materials for Milling	3.01
5. Prediction and Modelling of Thermic Processes & Fluid Production	2.56
6. Prediction and Modelling for COVID-19 & Medical Devices	2.37
7. Sentiment Analysis and Recommender Systems	2.37
8. Optimization of Job Scheduling	1.91
9. Gait Recognition and Modelling using Sensors, Wearables and Images	1.30

**Table 3:** Top 20 results of topic modelling inclusive of sub-topics.

Topic	% of Data
0. Security and Intrusion Detection	4.38
1. Fault Diagnosis and Detection	3.88
2. Forecasting Load and Power Demands	3.25
3. Industrial IoT and Wireless Communication	3.05
4. Tool wear, Cutting Parameters and Surface Materials for Milling	3.01
5. Prediction and Modelling of Thermal Processes and Fluid Production	2.56
6. Prediction and Modelling for COVID-19 and Medical Devices	2.37
7. Sentiment Analysis and Recommender Systems	2.37
8. Reinforcement Learning for Robotics in Assembly and Planning	2.00
9. Optimization of Job Scheduling	1.91
10. Modelling Control of Robotic Manipulators	1.83
11. Modelling for Processing Wastewater and Water Treatment	1.80
12. Predictive Maintenance and RUL Forecasting	1.63
13. Adaptive Motor Control	1.60
14. Quality Inspection	1.37
15. Fuzzy Risk Analysis and Safety Management	1.36
16. Pose Estimation for Grasping Objects	1.33
17. Gait Recognition and Modelling using Sensors and Images	1.30
18. Engineering Education and Skill Development	1.16
19. Forecasting Crop Yields	1.03

## Data Availability

The dataset generated during the current study is not publicly available as it contains proprietary information that the authors acquired through a license. Information on how to obtain it and reproduce the analysis is available in the presented work or from the corresponding author on request.
